# MixFit: Methodology for Computing Ancestry-Related Genetic Scores at the Individual Level and Its Application to the Estonian and Finnish Population Studies

**DOI:** 10.1371/journal.pone.0170325

**Published:** 2017-01-20

**Authors:** Toomas Haller, Liis Leitsalu, Krista Fischer, Marja-Liisa Nuotio, Tõnu Esko, Dorothea Irene Boomsma, Kirsten Ohm Kyvik, Tim D. Spector, Markus Perola, Andres Metspalu

**Affiliations:** 1 Estonian Genome Center, University of Tartu, Tartu, Estonia; 2 Institute for Molecular Medicine Finland, University of Helsinki, Helsinki, Finland; 3 Vrije University, Department of Biological Psychology, Netherlands Twin Register, Amsterdam, The Netherlands; 4 Department of Clinical Research, University of Southern Denmark, Odense, Denmark; 5 The Department of Twin Research & Genetic Epidemiology, TwinsUK Registry, Kings College London, London, United Kingdom; 6 National Institute for Health and Welfare, Helskinki, Finland; National Cheng Kung University, TAIWAN

## Abstract

Ancestry information at the individual level can be a valuable resource for personalized medicine, medical, demographical and history research, as well as for tracing back personal history. We report a new method for quantitatively determining personal genetic ancestry based on genome-wide data. Numerical ancestry component scores are assigned to individuals based on comparisons with reference populations. These comparisons are conducted with an existing analytical pipeline making use of genotype phasing, similarity matrix computation and our addition—multidimensional best fitting by MixFit. The method is demonstrated by studying Estonian and Finnish populations in geographical context. We show the main differences in the genetic composition of these otherwise close European populations and how they have influenced each other. The components of our analytical pipeline are freely available computer programs and scripts one of which was developed in house (available at: www.geenivaramu.ee/en/tools/mixfit).

## Introduction

Ancestry-related scientific questions are getting more attention due to the genome-wide and next generation sequencing data becoming available for growing number of individuals and populations [1,2]. This information is utilized in a variety of ways ranging from re-construction of historical events such as ancient migration patterns [3] to advancing personal and public health [4].

Knowing the ancestry information at the individual level can be essential in medicine as the disease risks and frequencies vary between the individuals of different ancestral groups [5]. Even relatively closely related populations display differences in allele frequencies and rare variation [2]. Knowledge of the genetic structure and variation of the study cohort is relevant in genome wide research [6].The research areas of human evolution, migration and demographics depend on accurate and efficient methods for obtaining ancestry information. Additionally there is a growing interest in the general public to learn about their own personal history as evidenced both by the public polls and the general wide-spread interest in commercial genetic information providers such as 23andMe [7,8].

Separating genetically relatively distant populations has been carried out with much success in numerous studies [9]. Several bioinformatics tools exist for these purposes, most notably Structure [10] and Admixture [11]. These methods rely on marker frequencies. The newer methods have gained in sensitivity by adopting haploblock-based approach and utilizing phasing. Phasing is commonly conducted with IMPUTE2 [12] or SHAPEIT [13]. ChromoPainter is a tool for mapping the genome with regard to the haploblocks while FineSTRUCTURE allows detailed study of the results [14]. These applications have been effectively combined to study fine population structure [15].

Tools for calculating individual ancestry are merging [1]. However, they often don’t use phased data and lack sensitivity to separate very close populations. We implemented a new analytical method for computing quantitative genetic ancestry components for individuals. The analytical pipeline combines existing free software solutions (SHAPEIT and ChromoPainter) together with an original script (MixFit). MixFit is our extension to the well established ChromoPainter output. It finds the best fit between the references and the individuals tested. Its outcome is numerical ancestry component assignments. In this work each unknown individual is assigned between three best-fitting reference groups as the optimal solution for a best fit method. The fractional membership is computed relative to each of the reference populations. The reliability of the fit is expressed as the fitting score, which measures the distance between the computed assignment and the maximally best fit. The fitting parameters are customizable (Section E–Section G in S2 File).

The MixFit output is fast to generate and straightforward to interpret. The novelty of our method is combining genotype data phasing and the resulting similarity matrices with multi-dimensional best fit. To our knowledge this approach has not been tested before for individuals and analyzed on a population-wide scale. Our method is sensitive to detecting small genetic differences as it allows separation of even genetically largely overlapping populations such as Estonians and Latvians. The computational steps are well documented and the ancestry assignments are easy to understand fractional memberships.

Estonian population serves as a good example of an ancestry study due to its small size and a good representativeness of a European population [16]. We discuss ancestry in the context of geography and compare the Estonian and Finnish populations.

## Materials and Methods

### Materials

All data were used confidentially and in accordance with all Estonian laws (EGRE) and University of Tartu regulations governing the use of genotype and phenotype data [17].Permission for this research was granted by the Research Ethics Committee of the University of Tartu.

We used the genome-wide data of the Estonian Genome Center, University of Tartu (EGCUT) (Section H in S1 File). The population-based EGCUT biobank maintains blood DNA samples and a multitude of associated phenotypic features (including demographic info) from all over Estonia [18,19]. Genotype information for the EGCUT cohort (Estonian cohort) was collected with Illumina Human OMNIExpress or 370CNV BeadChip genome-wide chips. Only high quality markers were used by applying the following quality control filters: call rate > 95%, MAF > 1%, HWE P-value >10^−6^. The Finnish Health 2000 population cohort (Finnish cohort) was used as a replication cohort [20] (Section I in S1 File). The samples were genotyped with Illumina Human610-Quad BeadChip.

The reference groups included 45 random individuals from each of the 22 European populations (Section G in S1 File). Data for 19 reference populations has been described [16,21]. These were genotyped with Illumina 370CNV BeadChip. The data for the 3 remaining references (Holland, UK, Denmark) were from the Genomeutwin study and have been described [22].

### Analytical pipeline

We deconvolute each individual's genetic ancestry components by combining existing bioinformatics tools with a best fit method. We use distance measures to study ancestry as opposed to the terms of clustering. These two ways of expressing identity and similarity are both valid.Our qualitative ancestry component determination analytical pipeline consists of SHAPEIT, ChromoPainter and MixFit. Practical details and instructions for using this pipeline are described (Section A–Section H in S2 File and Section A–Section C in S1 File). Briefly, these are the steps comprising the pipeline:

1. Compiling references (equal number of individuals for each reference) with the (unknown) individual studied.
2. Phasing reference individuals and the unknown individual together with SHAPEIT.
3. Similarity (chunkcounts) matrix creation with ChromoPainter. One matrix is created for all reference individuals, another one for the unknown together with the reference individuals. Therefore, for n unknown individuals n+1 matrices are created.
4. Compression of the matrices to reduce them to represent hypothetical “mean of all individuals” in the respective group.
5. Multi-dimensional best fit with the MixFit script to assign individual ancestry components to the unknown individual.

MixFit performs best fit between the references and the unknown individual to pick the reference populations and their relative ratios that best represent the unknown. The fractional representations of the references are called ancestry components. The “level of participation” in any given reference group for each individual is calculated considering the distances of all ancestry components as the reference groups cannot be described by a single distance measure. This allows us to separate very close reference populations as only some of their ancestry components may differ. It may be that some reference populations are sub-optimal or missing for a given unknown. In this case the second best solution is found and this is reflected accordingly in the assignment statistics. The less likely assignments should be removed from the study on the grounds that there were probably no good reference populations for that particular person.

The MixFit algorithm is customizable by selecting suitable parameters for the task (Section B-Section C in S1 File).

### Association analyses

To analyze statistical associations between the ancestry components and the phenotypic traits, classical linear regression was used for continuous traits (height) and logistic regression for binary traits (presence/absence of a certain eye color). All association analyses were conducted with the R software version 3.1.3 [23].

## Results

### Method validation

In this study chromosome 1 markers were used for computational feasibility. This chromosome was chosen because it proved as the best representative of the full genome (Section J in S1 File). The SNPs that were considered (about 19000) were common between all reference and unknown individuals.

The stability of the method was assessed as it includes a stochastic process (SHAPEIT). We determined overall consistency of 92% with experimenting with 20 individuals assigned 5 times independently (Section D in S1 File). Likewise the sensitivity to replication was assessed as we had 92 individuals genotyped with two different chips (Illumina Human OmniExpress and CNV370-DUO). The Pearson's correlation between the quantitative assignments of the main ancestry components with two chips ranged between 0.83 and 0.89 (Section E in S1 File).

The individual ancestry assignment validations are not straight forward because self-reported ancestry is not a continuous trait, nor can its extent be readily quantified. We nevertheless compared our assignments with the reported ancestry and demonstrated good fit (Section F in S1 File).

### Application to cohort studies

One method to assess the utility of the MixFit method is to transfer the individual-based ancestry assignments to the cohort level. We studied and compared the Estonian and Finnish cohorts.

#### Comparison of the estonian and finnish cohort

The Estonian and Finnish mean population ancestry component distribution suggested differences between the two neighboring nations (Table 1). While 88% of the ancestry components of the Finnish population were of Finnish (Finnish-south (FIN-S) or Finnish north (FIN-N)) origin, only 49% of the ancestry components of the Estonian population where of Estonian (EST) origin, thus suggesting greater heterogeneity among Estonians. The Estonian population contained a major Latvian (LAT) component (22.3%) and FIN-S component (13.1%) as well as a significant Russian (RUS, 7.4%) and Lithuanian (LIT, 5.6%) component. At the same time the foreign components of the Finnish population where smaller: Estonian (EST, 5.2%), Danish (DEN, 2.2%), Swedish (SWE, 1.3%).

**Table 1:** The main ancestry components for the Estonian and Finnish cohorts.

	CZH	DEN	EST	FIN-N	FIN-S	LAT	LIT	POL	RUS	SWE
**ESTONIA**		** **	** **	** **	** **	** **	** **	** **	** **	** **
mean (M)	0.009	0.002	**0.489**	0.005	0.131	0.223	0.056	0.003	0.074	0.005
mean-if-present (Mp)	0.331	0.052	**0.502**	0.087	0.257	0.305	0.175	0.436	0.245	0.336
Mx = M/Mp*100	2.8	2.9	**97.4**	5.6	50.7	73	32.1	0.8	30.2	1.3
count-if-present	284	294	**9712**	562	5062	7281	3201	80	3013	134
**FINLAND**										
mean (M)	0.004	0.022	0.052	**0.267**	**0.616**	0	0	0.001	0.004	0.013
mean-if-present (Mp)	0.238	0.091	0.104	**0.283**	**0.684**	0.117	0.019	0.34	0.1	0.185
Mx = M/Mp*100	1.7	24.1	49.9	**94.5**	**90.2**	0.1	0.2	0.3	4.2	7.2
count-if-present	33	464	963	**1823**	**1739**	2	4	6	81	138

Comparing the mean ancestry component values of all individuals (M) with the mean values of the individuals who had the particular component greater than zero (Mp) is indicative of genetic mixing (Mx = M/Mp*100) (Table 1). Estonian population shows a higher Mx value for the LAT component (73%) than the FIN-S component (50.7%), meaning that the LAT component is distributed more evenly in the Estonian population than the FIN-S component. This is in par with Estonia's long land border with Latvia as opposed to the sea border with Finland. Mixing between Estonians and Southern Finns has been the same in both directions as the Finnish population's Mx(EST) and Estonian population's Mx(FIN-S) are both around 50%.

#### Regional comparisons

We used self-reported Estonians (restricted to birth dates before 01.01.1970 –to reduce the effect of relocation) and Finns with two parents of self-reported Finnish origin in this study. We divided the individuals between regions based on the location where they were born. For the Finnish cohort only the birth places of the individuals' parents were known. We thus used both parent’s birth places for each individual, one half from each parent.

**Fig 1:**
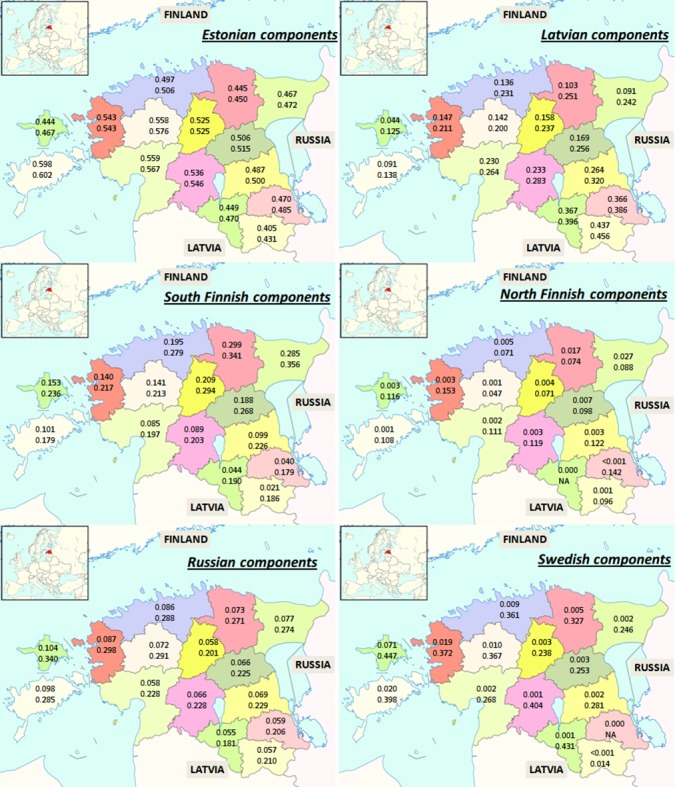
The mean values of four ancestry components for self-reported Estonians by the county. The top values represent the mean of all individuals (Ma), the bottom values represent the mean of the individuals who had the respective component value larger than zero (Mb).Background map image copyright University of Tartu, 2011.

Clear trends can be observed for the Estonian cohort linking individuals of various regions with the closest foreign country. The LAT component is the highest in the south-eastern counties near the border, showing a diminishing trend in the western and northern direction (Fig 1). This observation agrees with the historical fact whereby throughout the history Southern Estonia has been administratively more closely associated with the modern-time Northern Latvia than Northern Estonia [24]. The FIN-S component trend generally agrees with the land distance (and not the shortest distance) between Estonia and Finland. The SWE component is higher in the island of Hiiumaa (second largest Estonian island in the north-west—a location known to be with the strongest historical links to Sweden). The RUS component failed to show distinct gradients (Fig 1). We therefore show that geography corresponds well with ancestry and this supports the MixFit approach.

**Fig 2:**
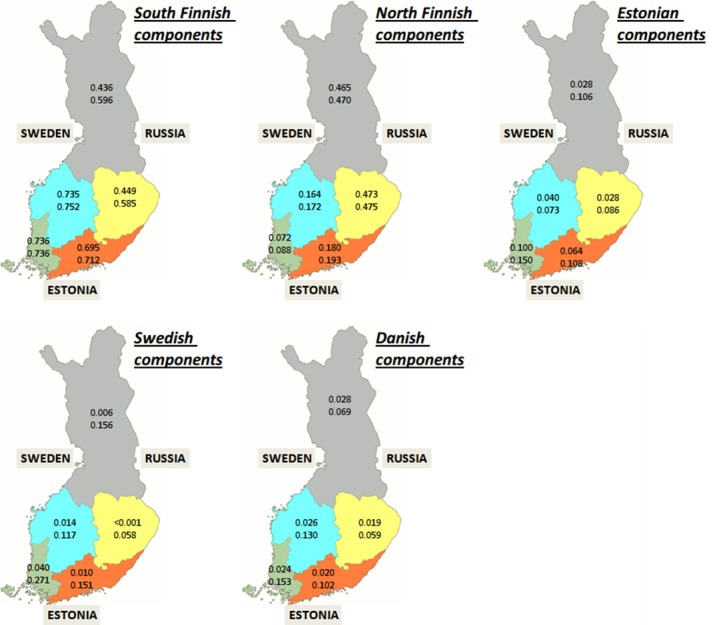
The mean values of the ancestry components for individuals with two Finland-born parents by the region. The top values represent the mean of all individuals, the bottom values represent the mean of the individuals who had the respective component value larger than zero.Background map image is a public domain image, reprinted from (https://commons.wikimedia.org/wiki/File:Suomi.karttapohja.2013.svg).

In the Finnish cohort the FIN-N component dominates in the north and FIN-S component dominates in the south (Fig 2). Interestingly the middle part of the country is divided vertically between these components: the eastern part is North-like and western part is South-like. The SWE component is most common in south-west—the region with strongest cultural association with Sweden. Surprisingly, the EST component is also most prevalent in the South-West, suggesting past interaction not by the land bridge (as was the case for the South Finnish component in Estonia) but via sea ways. This leads to a hypothesis suggesting that people have moved between Estonia and Finland in a clock-wise fashion: in the west the movement has been from Estonia to Finland while in the east the direction has been primarily the opposite.

The component values calculated for people who had the respective components greater than zero (the bottom values in the maps of Figs 1 and 2) indicate that the EST component is more evenly distributed among the people of south-west, and indeed the entire country, than the SWE component. Therefore, the genetic mixing with the Estonians is likely earlier than mixing with Swedes. This is so because if the component is more equally distributed it must have taken more time for it to reach that state. As two populations mix there is gradually going to be more people with the influences of the other population. After enough time the mixing is so through that the “foreign” component is very similar in most individuals i.e. the component is more equally distributed between individuals. Here, however, we must consider that our method may draw the line between the SWE and DEN components somewhat arbitrarily relative to the Finnish components. It may be more useful to view these two components collectively as a single “western” component when comparing them with the EST component.

#### Ancestry structure by the region–estonia

We studied the distribution patterns of the ancestry components by dividing Estonia into 5 regions: West, North, Middle, South-West and South-East (Section L in S1 File).

**Fig 3:**
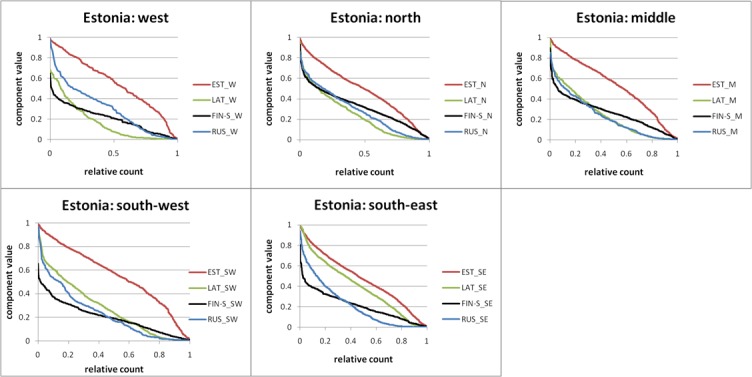
Ancestry components for 5 regions of Estonia. Only the individuals who had the respective ancestry components were considered. For each region all individual ancestry components were sorted in descending order. The x axis values were scaled to 1 and the points were connected by lines.

Decreasingly sorted (and x-axis values scaled to 1) ancestry components visualize the relative distribution of components of different magnitude (Fig 3). The EST component dominates all regions but the other components trade their dominance with regard to the other components significantly. The LAT component of the south-east mirrors the EST component very closely only with a smaller magnitude. No other components display this feature in any region. This argues for extremely close similarity between south-eastern Estonians and Latvians. In south-west the LAT component distribution and magnitude is more similar to that of the RUS component. The LAT component is very small in the west and also the north, the regions furthest away from Latvia. The LAT component prominence in the south-east as opposed to north-west reflects the political division of the time of Livonia–a medieval historical region that once melted today's Estonians and Latvians [24].

Although the FIN-S component dominates the foreign components of Northern Estonia its distribution is different from the LAT component in the south-east: its lower values are relatively less prominent. This argues for more recent mixing. The RUS component varies the least between different regions. In most regions its graph intersects the graph of FIN-S component thus suggesting later mixing with Russians.

#### Ancestry structure by the region–Finland

**Fig 4:**
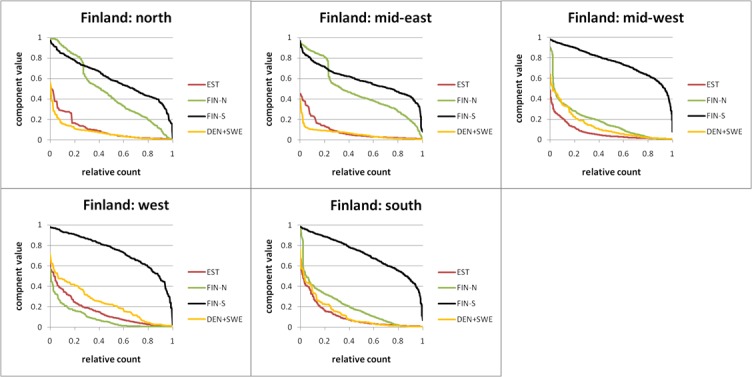
Ancestry components for 5 regions of Finland. Only the individuals who had the respective ancestry components were considered. For each region all individual ancestry components were sorted in descending order. The x axis values were scaled to 1 and the points were connected by lines.

The Finnish cohort individuals were divided between 5 regions based on the birth places of their parents (Fig 4). The northern and middle-eastern part of Finland have very similar profiles having both the FIN-N and FIN-S components in major quantities. The FIN-N component has a distinctive flat part in the high value regions indicating sub-populations that are genetically very isolated [25, 26]. In those regions the EST component dominates over the western components (SWE + DEN) and displays different mixing profile (more recent). The southern, western and mid-western parts of Finland form another region with the FIN-N component being comparable in magnitude to the foreign components. In the western part of the country the EST and western components dominate the FIN-N component. In the west and mid-west the western components prevail the EST component; however not so in Southern Finland. The relative mixing of the EST and western components in the south and west of Finland are quite similar (Fig 4).

#### Other comparisons of the estonian and finnish cohorts

**Fig 5:**
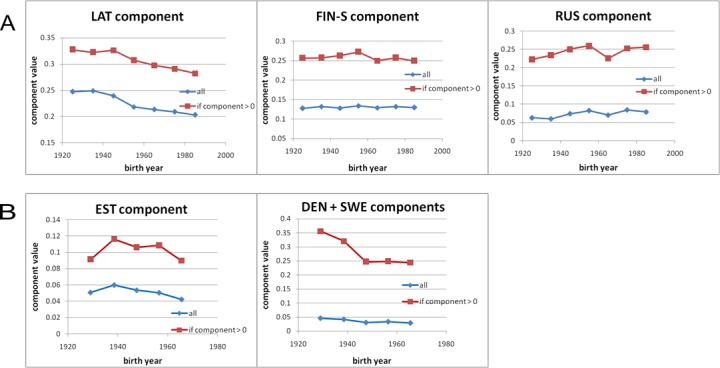
Temporal changes in ancestry components in Estonian (A) and Finnish (B) cohort.

It is of interest to population genetics and demographics fields to study the temporal dynamics of various components (Fig 5). In Estonia the LAT component has been on the decline since 1940ies. The Finnish components have been stable over the years observed. The RUS component, on the other hand, has been on a very slight upwards slope with a dip.

In Finland the introduction of western components was on the decline before 1940-ies and has stabilized as a flat plateau since then. The EST component is displaying a somewhat opposite trend (Fig 5).

#### Association studies with phenotypic traits

**Fig 6:**
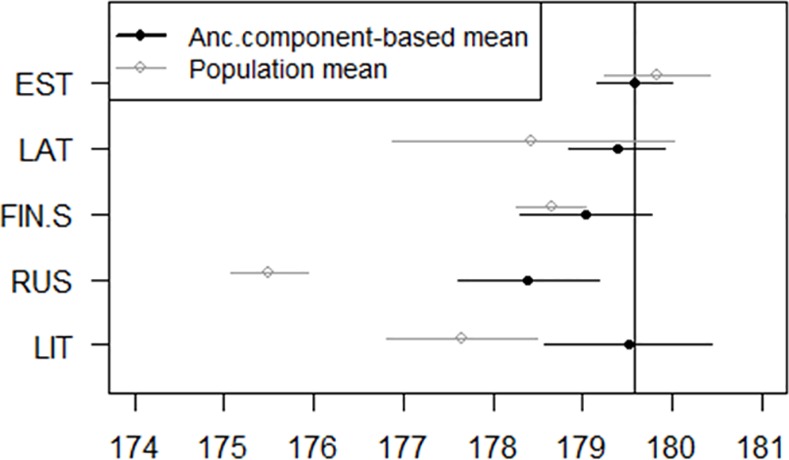
Ancestry components and height. Predicted mean height (with 95% Confidence Interval) for a 45-year old man whose ancestry component for one ethnicity was set to 1 and the others to 0 (based on linear regression modeling of the Estonian cohort), for the five most prevalent components in the Estonian cohort, compared to the observed population averages for Estonia, Latvia, Finland, Russian Federation and Lithuania (29).

**Fig 7:**
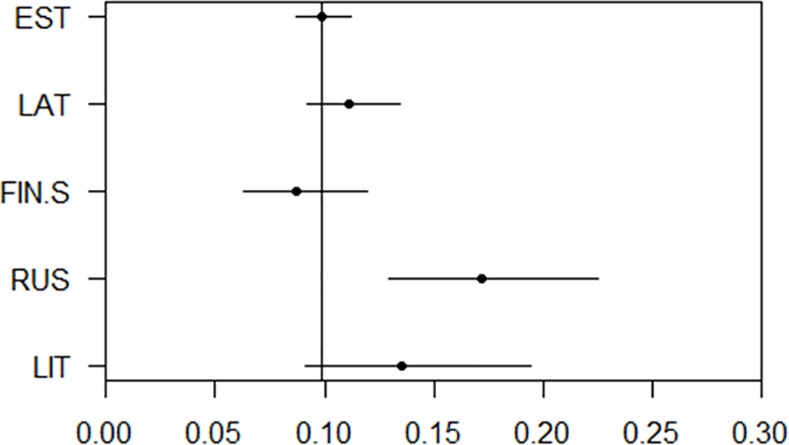
Ancestry components and eye color. Predicted probability of having brown eyes for an individual whose ancestry component for one ethnicity was set to 1 and the others to 0 (based on logistic regression modeling of the Estonian cohort data), for the five most prevalent components in the Estonian cohort.

**Fig 8:**
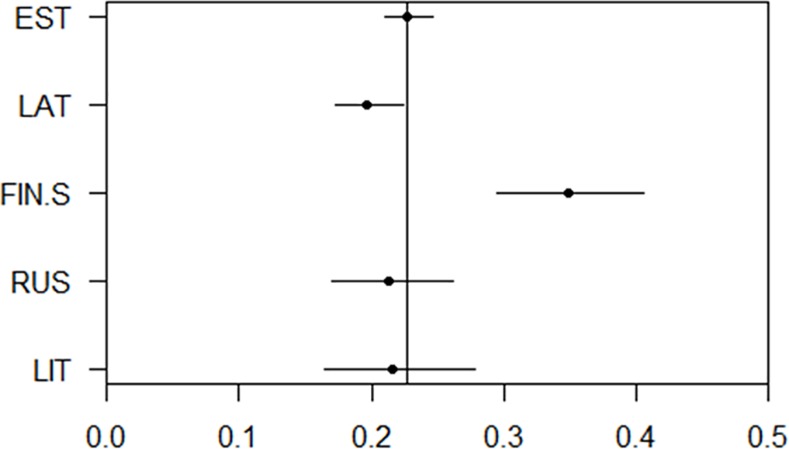
Ancestry components and hair color. Predicted probability of having blonde hair for an individual whose ancestry component for one ethnicity was set to 1 and the others to 0 (based on logistic regression modeling of the Estonian cohort data), for the five most prevalent components in the Estonian cohort.

Ancestry components can be used as continuous traits. We performed association studies with the five most prevalent ancestry components and anthropometric traits in the Estonian cohort. These studies were performed to demonstrate the utility of the ancestry components as measurable traits. We observed that the RUS and FIN-S component associated with shorter overall height (Fig 6). Additionally the FIN-S ancestry component associated with lighter eyes whereas the RUS and LIT components associated with a tendency to have brown eyes (Fig 7). The FIN-S component also had a significant association with lighter hair (Fig 8). We observed a general trend for lighter hair and eyes in the northern cohorts as opposed to the more southern ones.

## Discussion

In this work we present a new script that allows to assign ancestry components to individuals. This is done through the use of reference populations and with the help of pipeline using the SHAPEIT and ChromoPainter applications. The methodology is sensitive to the specifics of the references. Choosing the references is related to the questions that are asked. In this work we used individuals from different modern nations and defined ancestry as a degree of belonging to those groups (their multidimensional distance to the nations' cluster centers).

We attempted to deconvolute ancestry in today's geographical and political context so as to draw the connection between the ancestry trends and the current world. By doing so some of country-based reference groups were somewhat arbitrary in the genetic sense. However this is how the ancestry-related information was recorded when the reference groups were collected. We used the maximal number of reference groups at our disposal for the areas surrounding Estonia and Finland. We recognize that the reference set is not perfect, especially for for the eastern and southern regions (Ukraine, Belarus, Russia) and this needs to be considered when drawing conclusions.

Our reference groups contained 45 individuals. The size of the reference group can become an issue when the ancestry components of the unknown are all from very closely related references. On the other hand just a few individuals are needed for comparisons of distant populations. Most of the reference populations at our disposal were of size not significantly higher than 45 individuals; we therefore could not experiment with very large reference groups.

The wide range of birth years was addressed by restricting the birth date to 1970. This optimization step excluded younger people (whose birth locations may not be very connected to their ancestry) and at the same time retained enough subjects to study. Based on the demographical trends of Estonia we believe that people were sufficiently conservative about moving to new locations for childbirth before the 1970's. Additionally, people of different age are more or less equally distributed about the country and we therefore do not expect major age effects.

We evaluated the algorithm and implementation by studying the ancestry components at the population (cohort) scale. This route was selected as we did not have enough individuals with well documented foreign ancestry in either of the two cohorts. As ancestry as such is always a disputable characteristic, it cannot be directly measured to easily evaluate the accuracy of the algorithm. Showing its use at the population scale was the best method currently at our disposal. However, we rather expect the main use of our approach to be solving ancestry questions for specific individuals.

The study of the Estonian and Finnish cohort allowed us to show the utility of our ancestry analysis method but also lead to findings that describe the history of the two nations. These discoveries need to be followed up by separate studies so that the hypotheses could be independently confirmed. However, we were able to quantify the distribution of foreign influences about the countries. We showed that they were in good accordance with geographical distances and historical events. For example, South-Eastern Estonia has maintained its genetic ties with Latvia in the south. This is in contrast with South-Western Estonia which is equally close to Latvia but has spent less time in history being in the same administrative unit with Latvia and also has less population density along the border area. It came somewhat unexpected that the Latvian influence decreased in such a monotonous and convincing way as a function of north-west to south-east distance. Also, the Latvian influence as a function of this distance has a rather steep gradient. We also showed that the Finnish influence in the Estonian cohort follows the land distance rather than the distance through the main modern connection point: Tallinn, Estonia–Helsinki, Finland sea line. This indicates that the mixing influences seen are not very recent.

The Russian influence was minor in both the Estonian and Finnish cohorts. The Russian reference in the current study is likely under-estimated as it came entirely from the Tver region of Russia and was therefore not fully representative of Russia. Large countries should be divided into sub-regions because of the great magnitude of the genetic variation [27].

We observed a proportionally rather large DEN signal in the Finnish cohort which was surprising. We currently suspect that the SWE and DEN component signals may merge/interfere when comparing them with the EST component signals. We therefore treated the DEN and SWE components collectively as “western” components.

Regional dissections of the ancestry components allowed us to make several interesting observations. First of all, even a country as small as Estonia and without apparent geographic and cultural barriers has a significant structure in the genetic landscape [16]. The regional studies also revealed a specific nature of the FIN-N component in the Finnish cohort. This component was present in relatively large quantities in the northern and eastern regions. We believe that using two distinct Finnish components for Finland (FIN-N and FIN-S) is justified by the large land area differences between the two countries. It is well documented that Northern Finnish and Southern Finnish populations are genetically different [26]. We acknowledge that the results are influenced by the fact that two Finnish ancestry component types were used.

Based on the results we hypothesize that at some point in history the migration of the ancestral identity between Estonia and Finland has taken place in a clock-wise fashion, being in the southerly direction in the east (via land bridge) and northerly direction in the west (via sea ways). However, this observation may be a result of two opposing trends taking place at different time periods and having different lengths and magnitudes. The simple conclusion is also complicated by the “washing out” effects caused by the other parallel migration trends of other ancestry components which probably have been both qualitatively and quantitatively different between east and west. The temporal studies (Fig 5) suggested that mixing rates between the neighboring populations have not been the same even through the most recent past.

We performed association studies between the ancestry components and several anthropometric traits using the Estonian cohort. Our studies were limited by power and presence of enough foreign ancestry components in the cohort. Nevertheless, we detected several associations that were statistically significant. We report here, the body height, hair color and eye color as the relevant findings in association with the ancestry components. Our findings agree with the general tends observed for the European populations [28].

The differences between the ancestry component-specific mean heights are similar to the differences between mean heights in the countries where the corresponding ethnicity is dominant (Fig 6). The RUS ancestry component is associated with significantly lower average height, but the reported average height in the Russian Federation is even lower than the prediction according to the ancestry component [29]. The possible reason is that the RUS reference sample did not cover the whole country and is sampled from a region that is geographically close to Estonia.

The effect of the FIN-S component on body height is interesting. We hypothesize this to be the influence of shorter height of Northern Finland. However, genetic determinants of height have been extensively studied [30] and there have been observations that individuals in northern Finland are shorter than those in the south [31].

## Strengths and Limitations

Our analytical method has certain limitations. As the definition of nationality is ambiguous and can be understood in multiple ways the input data (i.e. reported nationality) quality is the main factor influencing the outcome. The reference populations need to be representative of all ancestry components of the individual and they need to represent each nation in a uniform way–neither of these is trivial to achieve. For better comparison larger countries should be broken down into regions as a small number of individuals cannot be as adequately representative of those countries as they can be of smaller countries.

We predict that the general methodology also applies when the populations are more mixed. In that case obtaining the relevant reference populations becomes very important. The reference populations should be quite uniform in terms of their ancestry. Alternatively one could divide them into several sub-references or, if a more general trends are studied and sensitivity is not a major concern, then growing the size of reference populations can also help.

We acknowledge that the population (cohort) level evaluation is not most optimal for evaluation of our algorithm. However, this approach was considered the best option in the given situation. The population level trends can give a good sense of the value of the algorithm. There is no “gold standard” approach when it comes to measuring the correctness of ancestry assignment as different methods usually give at least somewhat different results. Even when the ancestry of a given person is known rather accurately it is usually so only in the geographical and cultural sense of the word, rather than genetic.

Since we are using mathematical best fit where all reference populations “mathematically compete for their share in the ancestry profile” it is not easily possible to assign more than a small number of ancestry components to each person. To aid in estimating the plausibility of the assignments, MixFit calculates several parameters that allow to evaluate the statistical quality of each assignment.

The current pipeline is making use of several applications and is therefore modular. This nature allows easy replacement of individual steps. For examples similarity matrices can be created for MixFit using other methods. However, we believe that our current approach is optimal as it ensures the required sensitivity to discriminate between closely related populations. We see value for it in the real life situations when determining ancestry or when another method for determining ancestry is needed for a “second opinion”.

## Supporting Information

S1 File Analytical pipeline used (Section A). MixFit algorithm (Section B). Explanation how MixFit algorithm works (Section C). Stability of the method (Section D). Sensitivity to replication (Section E). Method validation–comparison with self-reported ancestry (Section F). Reference populations used (Section G). Estonian cohort description (Section H). Finnish cohort description (Section I). Chromosome selection (Section J). Year of birth distribution (Section K). Regions of Estonia (Section L). Estonian cohort acknowledgments (Section M). Finnish cohort acknowledgments (Section N).
(PDF)

S2 File Data preparation (Section A). Phasing with SHAPEIT (Section B). Chromosome painting with ChromoPainter (Section C). Chunkcounts matix manipulations (Section D). MixFit analysis (Section E). Testing (Section F). MixFit output file (Section G). Computational speed (Section H).
(PDF)
