# Supplementary material for: MixFit: Methodology for Computing Ancestry-Related Genetic Scores at the Individual Level and Its Application to the Estonian and Finnish Population Studies
Source: PLoS One. 2017 Jan 20;12(1):e0170325. doi: 10.1371/journal.pone.0170325 (PMC5249084; doi:10.1371/journal.pone.0170325)
Supplement: S1 File — Analytical pipeline used (Section A). MixFit algorithm (Section B). Explanation how MixFit algorithm works (Section C). Stability of the method (Section D). Sensitivity to replication (Section E). Method validation–comparison with self-reported ancestry (Section F). Reference populations used (Section G). Estonian cohort description (Section H). Finnish cohort description (Section I). Chromosome selection (Section J). Year of birth distribution (Section K). Regions of Estonia (Section L). Estonian cohort acknowledgments (Section M). Finnish cohort acknowledgments (Section N). (PDF) [file pone.0170325.s001.pdf]

Supplementary Materials

Section A. Analytical pipeline used

Below are the required steps to use the ancestry pipeline. The technical details are found in the Practical Analysis Guide.

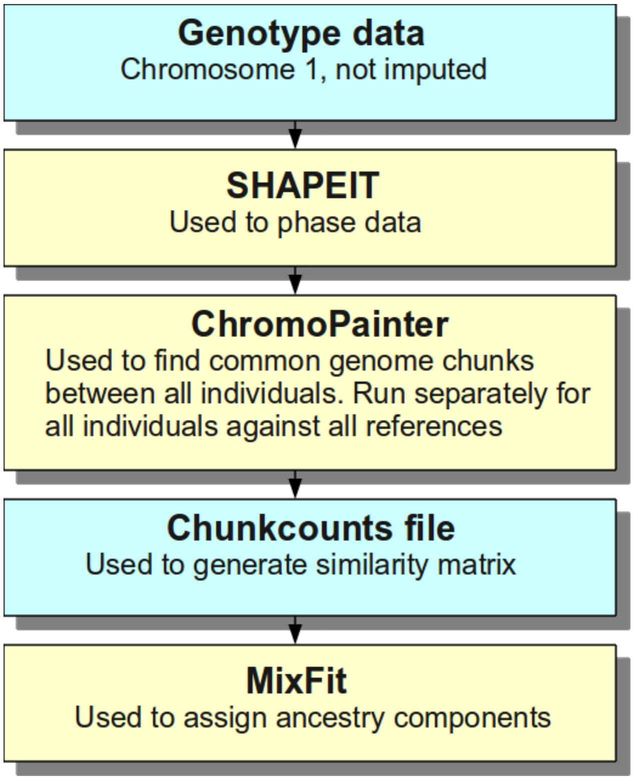

1. Reference individuals' genome wide data were compiled in ped/map files (Plink format) so that each ancestry reference group (22 total) was represented by the same number of individuals (45, limit determined by the smallest size reference group) based on self-reported ancestry. The unknown individual's data were appended to the end of the reference file.

2. Compiled genotype data were phased with SHAPEIT. The results were converted into the IMPUTE2 format for subjecting to the following ChromoPainter step (script from the ChromoPainter website).

3. ChromoPainter was used to divide the phased genome data into chunks based on genetic identity. The resulting chunkcounts file is a matrix which lists pair-wise similarity between the individuals in terms of the number of common genome chunks. Each genome chunk is always assigned to the best fitting individual pair. This means that all individual pairs “compete” for the chunk assignments and as a result it is important that each unknown data set experiences the same “neighbors” (is combined with the same reference data sets) during the chunk assignment process. Each ChromoPainter run produced an array of 991 numbers (ARRAY) indicating that particular individual's similarity with all 990 reference individuals and itself. The same ChromoPainter analysis was also repeated for all references in the absence of any unknowns so that a matrix of 990 x 990 numbers (MATRIX) is produced showing every reference individual's similarity with all other reference individuals.

4. Chunkcount matrix manipulations. The ARRAY contains counts of common chunks between the unknown individual and the reference individuals. Each reference belongs to one of the reference group (nationality group). The common chunk counts of all references are averaged within each reference group for the unknown. As a result, the individual is characterized by their similarity with each reference group as a whole (via a "hypothetical average person") and no longer with each reference individual separately. This horizontal compression reduces the number of columns in the matrix to that of the reference groups (22). The same horizontal compression is also carried out for the MATRIX. Since the MATRIX contains the same individuals both horizontally and vertically, it is additionally compressed vertically following the same logic. The resulting MATRIX has both dimensions equal to the number of the reference groups and each value represents the average number of common chunks between the two reference groups. The reference groups in the MATRIX are now expressed in the same way as the individuals in the ARRAY. The ARRAY and the MATRIX are additionally scaled across the columns so that the mean value in each row becomes equal to one. These steps create genetic similarity matrices a) between the unknown and each reference population, b) between each reference population and all other reference populations. Note that the described matrix manipulations can be performed with MS Excel or similar software.

5. MixFit analysis. MixFit finds the mathematical best fit between the ARRAY and the rows of the MATRIX to determine the mix (amalgamate) of references that best describes the unknown in terms of the normalized average common chunk distribution. Three best-fitting references (rows of the MATRIX) are identified and quantified for each unknown. The fractional values of the references that best explain the ancestry of the unknown are called the ancestry components. The best three ancestry components are determined by considering all combinations of all references. This "amalgamate" deconvolution is similar to deconvolution of composite color into individual RGB components (example: green = 50% blue, 50% yellow, 0% red). The MixFit fitting process is a multi-dimensional fitting where similarity between an individual and a reference group is considered maximal when the sum of all sub-distances linking the individual and the reference is minimal. The sub-distances are those between the ancestry components of the individual and a reference and are expressed as group-averaged and scaled common genomic chunk counts. With 22 reference groups, the sum of all 22 distances between the individual and all references would have to be minimal. As an example, a person is more likely to belong to a hypothetical Group A not if the genetic identity (chunk count) with the average Group A individual is the highest but when all of its distances from the other groups are as similar as possible to those between Group A and the other groups (this is further explained below). Therefore the distance between two groups is not defined simply as the distance between certain genetic ancestry components but a global best fit of all ancestry components considered. This approach enables to better deconvolute the ancestry components because the distances are not simply linear measures but rather locations on a multi-dimensional landscape.

Section B. MixFit algorithm

The MixFit application allows to change various assignment settings and therefore modify the assignment algorithm (usually to respond to the training sets most adequately). The algorithm used in this work is as follows:

MixFit isolates up to 3 reference populations that collectively most closely resemble the composition of the unknown. Initially there are total of n reference populations. All references are tested (3 at a time) against the others by gradually changing their relative ratios in the mixture of 3 references and comparing the result with the unknown.

As the reference fractions are systematically varied relatively one another three at a time (ONE is varied from 0 to 1, TWO is varied correspondingly from 1 to 0 and THREE is held constant; then the same logic is repeated for a new value of THREE) the fit between the mixture and the unknown fluctuates smoothly between better and worse. The local best fit minima are detected and their corresponding reference ratio values are saved. The values that were among the best 30% (this can be changed with the "–a1" flag followed by a number between 0 and 1 in the command line) of minima values are kept for the following steps.

When all reference combinations are tested (in increments of 0.01, this can be changed) all reference ratio values from all runs that ranked among the 20% best ones (in terms of fit with the unknown; from among the ones that passed the "–a1" flag filter) are added together by the reference. (This can be changed with the "–a2" flag followed by a number between 0 and 1 in the command line). Now each reference has a value that indicates how much it was "needed" in all simulations to achieve the best fit. The references are ranked according to these scores and the three highest ranking references are the ancestry components for the unknown. Because the three components may have all come from unrelated simulation runs, one more simulation is performed to find the best ratios among the three chosen references. For this a combinatorial simulation is carried out such that all ratios of all three references are tested against the unknown. The best 10% of the values (this can be changed with the "–a3" flag followed by a number between 0 and 1 in the command line) are averaged for the final answer of what the best ratio between the three references is expected to be.

Section C. Explanation how MixFit algorithm works

Below is a simplified example of how MixFit algorithm computes distances and assigns components. Genetic similarity values of the references and the Unknown are represented as values with the mean scaled to one. GOF (goodness of fit) scores are determined for the Unknown relative to each reference as shown:

	Compone	Compone	Compone	sum of columns (GOF score)
**Ref A**	**1.4**	**1.1**	**0.5**	
**Ref B**	**1.1**	**1.6**	**0.3**	
**Ref C**	**0.8**	**1.2**	**1**	
**Unknown**	**1.3**	**1.3**	**0.4**	
**abs(Unknown - Ref A)**	0.1	0.2	0.1	**0.4**
**abs (Unknwon - Ref B)**	0.2	0.3	0.1	**0.6**
**abs (Unknown - Ref C)**	0.5	0.1	0.6	**1.2**

The GOF score suggests that the references can be ordered as Ref A, Ref B, Ref C according to their similarity with the Unknown.

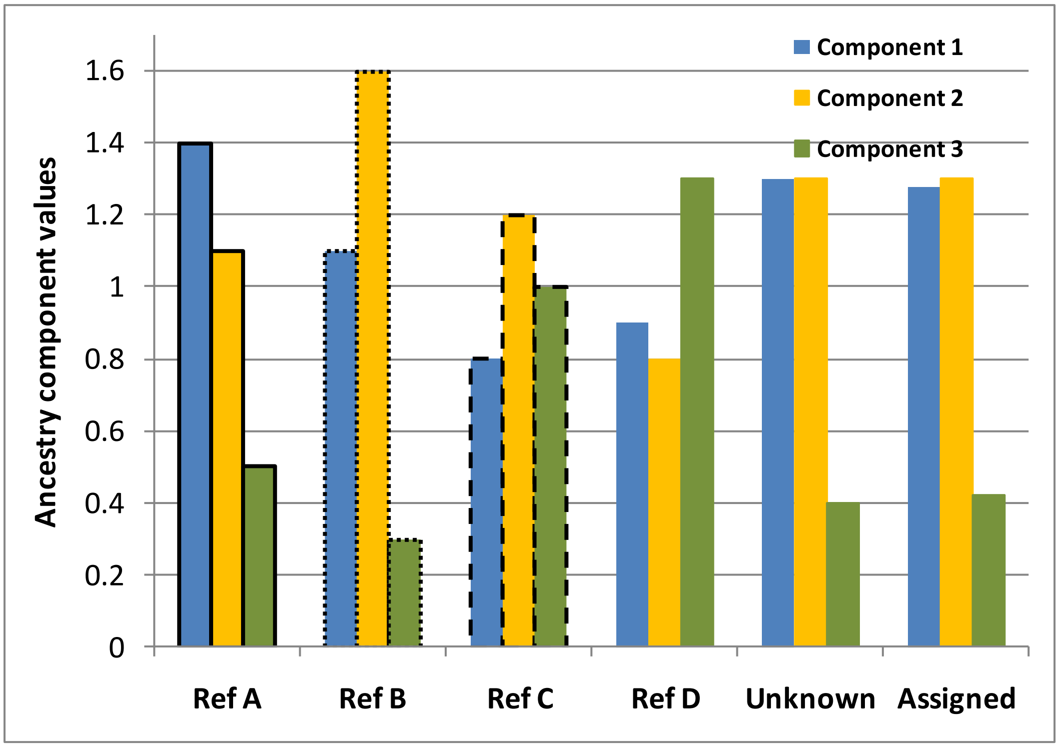

The amalgamate of Ref A and Ref B shows the following origin of the three components (bar border styles are the same as in the figure above):

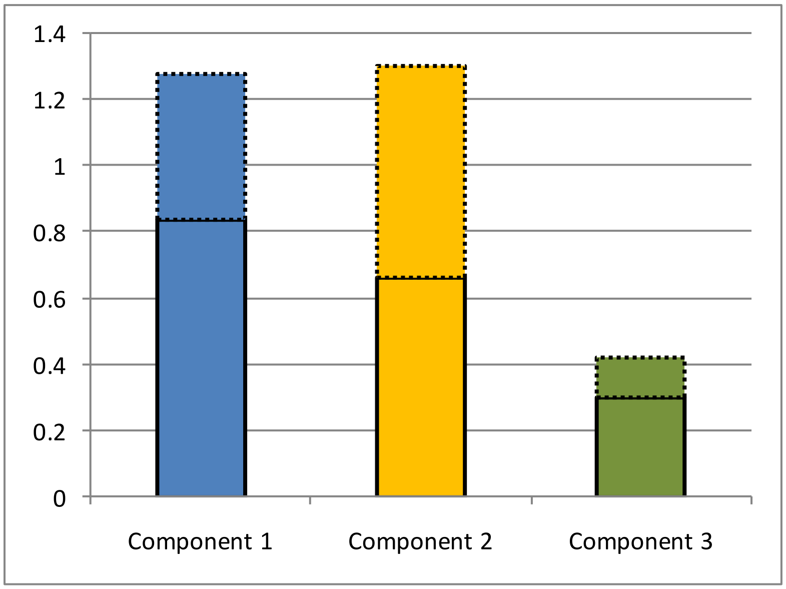

While 60% of the components originate from Ref A, about half of the component 2 is derived from Ref B (due to the larger component 2 signal of Ref B).

Section D. Stability of the method

We determined the stability of our pipeline by analyzing a subsection of individuals multiple times. Twenty randomly selected individuals (self-reported Estonians) were selected and analyzed with the SHAPEIT-Chromopainter-MixFit pipeline 5 times. SHAPEIT is a stochastic process and introduces variation. The amount of variation was quantified. The main ancestry component assignment (considering 5 assignments for each individual) was used as the reference and the discrepancies from it were found (among the 5 assignments).

Of the 20 individuals 15 were assigned identically all 5 times. Three individuals had one discrepancy (one ancestry component was assigned differently) in one of the five assignments (misses=3*1), 1 individual had 1 discrepancy in 2 of the 5 assignments (both were the same; misses=1*2), 1 individual had one discrepancy in 3 of the 5 assignments (two different types; misses=3*1). The overall constancy therefore is estimated as the number of misses relative to all assignments: (20*5) - 8 = 92%. In all cases (within each set of 5 assignments) two of the three ancestry components were always the same. Average variance of assignment of the major ancestry component across all 20 individuals was 0.0072.

Section E. Sensitivity to replication

Some individuals (92) were genotyped with two chips (Illumina Human OmniExpress and 370CNV). This offered an opportunity to determine ancestry assignment variation in real situations. When going from OmniExpress to 370 CNV the mean Estonian component of the Estonian cohort changed from 0.550 +- 0-304 to 0.569+-0.307 (Pearson's correlation = 0.83) while the Latvian component changed from 0.189 +-0.286 to 0.179 +-0.274 (Pearson's correlation = 0.89). Given the small number of markers used (19000) and the variation in genotyping, these results were found highly acceptable.

Section F. Method validation – comparison with self-reported ancestry

The EGCUT database individuals primarily self-categorize themselves as Estonians. However, it also has a small number of other nationals. Of the 27 self-reported Finns all but 2 (92.6%) had major Finnish components (defined here as larger than 0.2) detected while only 17.6% of self-reported Estonians had Finnish components over 0.2; 39% of Estonians had at least some Finnish component (>0.01). The mean Finnish component for the 27 self-reported Finns was 0.73+-0.33 (South Finnish 0.63+-0.29, North Finnish 0.1+-0.14), while the same for self-reported Estonians was 0.09+-0.17 (South Finnish 0.08+-0.17, North Finnish 0.01+-0.03). Because Estonia is geographically closer to the southern border of Finland, the larger South Finnish component matches the expectations.

The data from the Health 2000 (Finland) used in this study contains self-reported nationality information for 2000 individuals. Among them 10 individuals have two (self-defined) foreign parents. We analyzed these individuals to determine the fit with the computed ancestry (Table 2). All individuals had at least one component belonging to the group of one of the parents, or a group geographically directly neighboring a group of one of the parents.

ID	comp1	value1	comp2	val2	comp3	val3	Z-score (GOF)	parent1	parent2
ind1	**GER-N**	**0.7**	**HOL**	**0.3**	NA	NA	-1.05	GER	GER
ind2	**EST**	**0.57**	**LAT**	**0.28**	**FIN-S**	**0.15**	0.03	EST	EST
ind3	**EST**	**0.69**	**LAT**	**0.21**	LIT	0.1	-0.66	EST	EST
ind4	**POL**	**0.84**	**EST**	**0.16**	NA	NA	-1.10	GER	LAT
ind5	**HOL**	**0.96**	ITA-S	0.04	NA	NA	-0.87	HOL	HOL
ind6	**DEN**	**0.87**	ITA-N	0.13	NA	NA	-1.62	GER	GER
ind7	**CZH**	**0.82**	SWE	0.18	NA	NA	-1.05	GER	GER
ind8	**POL**	**0.66**	BUL	0.34	NA	NA	-0.49	POL	POL
ind9	POL	0.56	**LAT**	**0.44**	NA	NA	-0.69	EST	EST
ind10	**SWE**	**0.89**	**FIN-S**	**0.11**	NA	NA	-0.06	NOR	NOR

Table. Data for 10 individuals with two foreign parents from the Health 2000 cohort (Finland). Self-reported nationality is shown in the last two columns. The components representing the country or neighboring country of either of the parent are shown in bold.

Section G. Reference populations used

The reference populations included 45 randomly selected individual from each population. The number of individuals used was dictated by the size of the smallest reference set as it is important to have the same number of individuals in each reference group. The reference groups were defined as: Austria (AUT), Bulgaria (BUL), Czech Republic (CZH), Denmark (DEN), Estonia (EST), Finland north (FIN-N), Finland south (FIN-S), France (FRA), Germany north (GER-N), Germany south (GER-S), Holland (HOL), Hungary (HUN), Italy north (ITA-N), Italy south (ITA-S), Latvia (LAT), Lithuania (LIT), Poland (POL), Russia Europe (Tver) (RUS), Spain (SPA), Sweden (SWE) Switzerland (SUI), United Kingdom (UK).

Section H. Estonian cohort description

The Estonian Biobank is the population-based biobank of the Estonian Genome Center of the University of Tartu (EGCUT). The project is conducted according to the Estonian Gene Research Act and all participants have signed broad informed consent (Metspalu 2004, Drug Dev. Res., Leitsalu 2014, Int. J. Epidemiol.). The cohort size is currently 51535 people from 18 years of age and up. All subjects are volunteers and were recruited randomly by general practitioners (GP) and physicians in hospitals. A Computer Assisted Personal Interview is conducted at the doctor's office to record personal data (place of birth, place(s) of living, nationality etc.), genealogical data (family history spanning four generations), educational and occupational history, lifestyle data (physical activity, dietary habits - FFQ, smoking, alcohol consumption, women's health, quality of life). The UTARTU database has been linked with the national registries and hospital databases for obtaining up-to-date phenotypic information. The law enables re-questioning and the rules to access data and samples are clear and transparent (http://www.geenivaramu.ee/en/access-biopank/data-access).

Further cohort description:
Leitsalu L, Haller T, Esko T, et al. Cohort Profile: Estonian Biobank of the Estonian Genome Center, University of Tartu. Int. J. Epidemiol. 2014. Available at: http://www.ncbi.nlm.nih.gov/pubmed/24518929. Accessed June 27, 2014.

Section I. Finnish cohort description

Health 2000 is a population-based national survey on the health and functional capacity of Finnish individuals (http://www.terveys2000.fi/julkaisut/baseline.pdf). The main aim of the survey is to study the prevalence and determinants of the most important health problems and the associated need for care, rehabilitation and help among the working-aged and aged population. A nationally representative sample of 10,000 individuals was drawn of the population aged 18 years and older. The results of this study are used for examining trends in national health as well as for research purposes. The survey included an interview about medical history, health-related lifestyle habits, and a clinical examination (for individuals of 30 years of age and older) at which a blood sample was drawn. A detailed description of the study protocol is available at http://www.terveys2000.fi/doc/methodologyrep.pdf. Study participants were followed up through 31 December 2010 and restricted to be aged ≤80 years at baseline. In this, the GenMets sample that has been described in detail previously (Kristiansson et al.), was used. Individuals in GenMets are metabolic syndrome cases and matched controls drawn from the Health 2000 study.

Genotyping platform & SNP panel: Illumina Human 610K BeadChip, Genotype Calling software: Illuminuns, Genotyping center: Sanger Institute (Cambridge, UK). Genotyping QC: Exlusions (subject): Call rate <95%, other: heterozygosity, gender check and relatedness checks. Exlusions (SNP): call rate <95%, MAF <1%, HWE 1*10-6.

Further cohort description:
Kristiansson K, Perola M, Tikkanen E et al. Genome-wide screen for metabolic syndrome susceptibility loci reveals strong lipid gene contribution but no evidence for common genetic basis for clustering of metabolic syndrome traits. Circ Cardiovasc Genet 2012;5:242-249.

Section J. Chromosome selection

An ancestry assignment experiment was carried out with data from 22 autosomal chromosomes. We compared the results from individual chromosome experiments with those of the whole-genome analysis to show that chromosome 1 alone served as the best chromosome to represent the whole genome. We concluded that in the interest of computational feasibility ancestry assignments can be based on chromosome 1 alone.

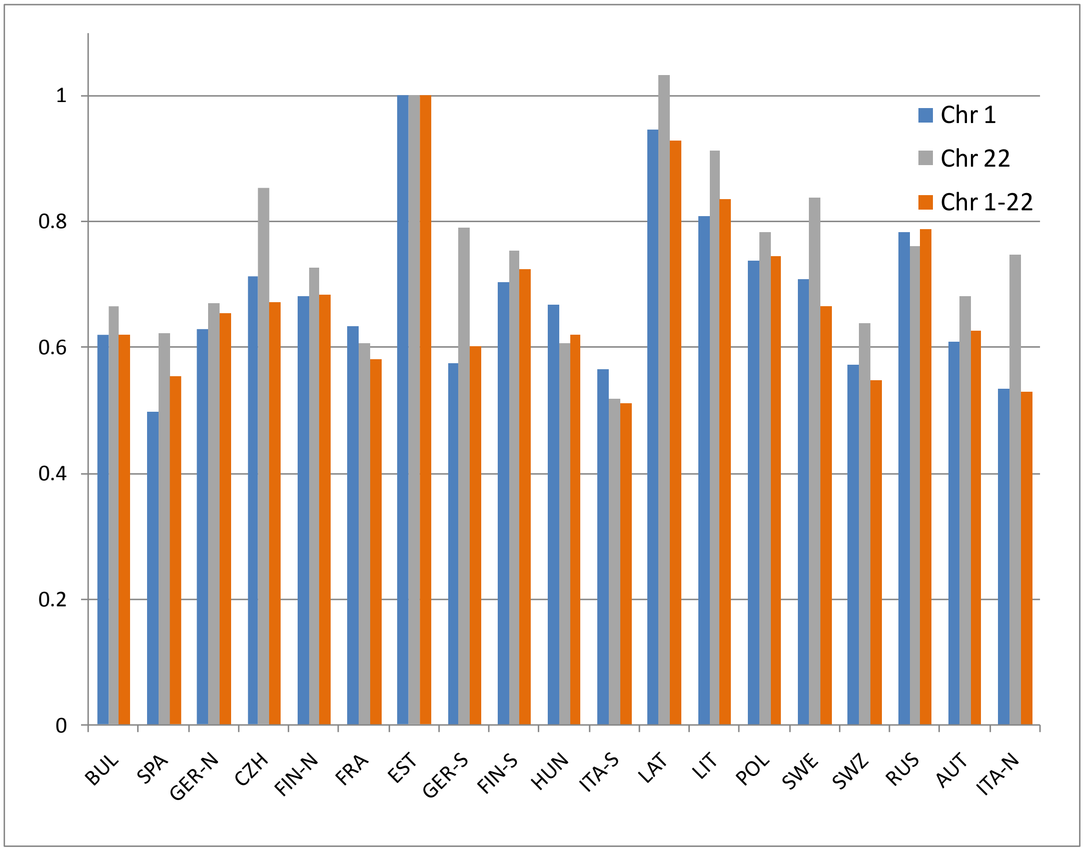

Fig. S1. A representative example showing ancestry assignments for one individual with chromosome 1, chromosome 22 and the mean of all 22 autosomal chromosomes. Chromosome 1 results were most similar and chromosome 22 results were least similar to those of the mean value of all autosomal chromosomes. Results were scaled with respect to the EST component.

.

Section K. <u>Year of birth distribution</u>

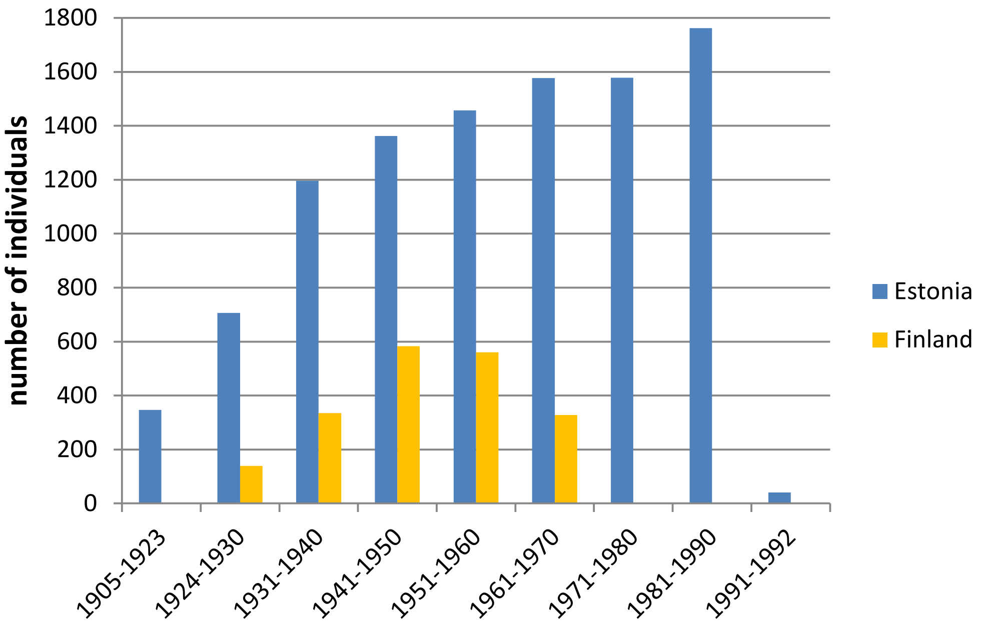

Fig. S2. Histogram of year of birth for 10026 self-reported Estonians and 1945 Finns with two Finland-born parents.

Section L. Regions of Estonia

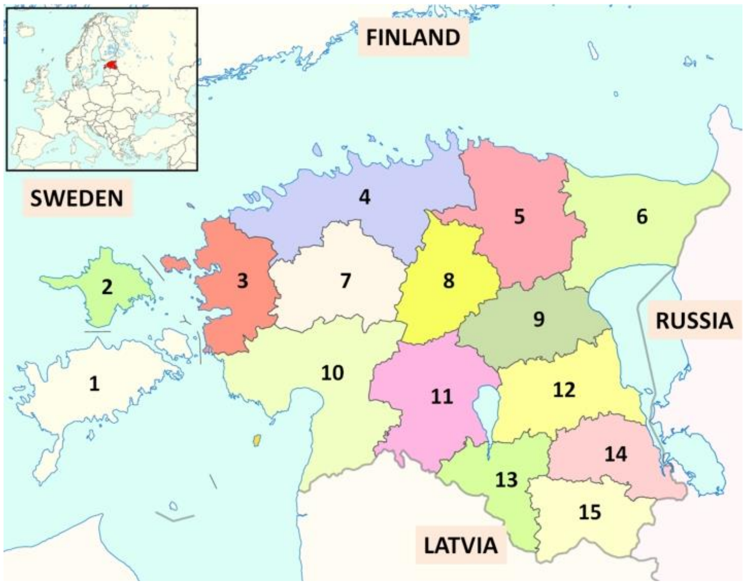

Fig. S3. Dividing Estonia into regions: a) West; counties 1,2,3, b) North; counties 4,5,6, c) Middle; counties 7,8,9, d) South-West, counties 10,11, e) South-East; counties 12, 13,14,15.

Section M. Estonian cohort acknowledgements

EGCUT received support from EU FP7 grant BBMRI-LPC (#313010), H2020 grant ePerMed (#692145), targeted financing from Estonian Government IUT20-60, IUT24-6, Estonian Research Roadmap through the Estonian Ministry of Education and Research (3.2.0304.11-0312), Center of Excellence in Genomics (EXCEGEN), This work was also supported by the US National Institute of Health [R01DK075787].

Section N. Finnish cohort acknowledgements

The Health 2000 Study was funded by the National Institute for Health and Welfare (THL), the Finnish Centre for Pensions (ETK), the Social Insurance Institution of Finland (KELA), the Local Government Pensions Institution (KEVA) and other organizations listed on the website of the survey (http://www.terveys2000.fi). M.P. is partly financially supported for this work by the Finnish Academy SALVE program ‘‘Pubgensense’’ 129322 and by grants from Finnish Foundation for Cardiovascular Research.
