# Supplementary material for: MixFit: Methodology for Computing Ancestry-Related Genetic Scores at the Individual Level and Its Application to the Estonian and Finnish Population Studies
Source: PLoS One. 2017 Jan 20;12(1):e0170325. doi: 10.1371/journal.pone.0170325 (PMC5249084; doi:10.1371/journal.pone.0170325)
Supplement: S2 File — Data preparation (Section A). Phasing with SHAPEIT (Section B). Chromosome painting with ChromoPainter (Section C). Chunkcounts matix manipulations (Section D). MixFit analysis (Section E). Testing (Section F). MixFit output file (Section G). Computational speed (Section H). (PDF) [file pone.0170325.s002.pdf]

Practical Guide to the Analytical Pipeline

This guide uses the numbers (of individuals) used in this study. The numbers can be freely changed according to each specific study design.

Section A. Data preparation

Genotyping was done with Illumina Human OMNIExpress and 370CNV-DUO and formatted to PLINK ped/map files (data.ped, data.map). Known references' (22 total, 45 individuals in each) genome-wide data for chromosome 1 were combined with those of the unknown so that the unknown appears last in the ped file. Only the markers that were common between all individuals were selected (about 19000). Data manipulations were performed with PLINK (http://pngu.mgh.harvard.edu/~purcell/plink/) and GTOOL (http://www.well.ox.ac.uk/~cfreeman/software/gwas/gtool.html).

Note: It is important that a) each reference group contains data for the same number of individuals, b) each unknown data set is combined with the same references data sets for accurate comparisons of the unknowns' results later in the pipeline.

Section B. Phasing with SHAPEIT

Phasing and conversion to haps/sample with SHAPEIT (www.shapeit.fr):

```
SHAPEIT --input-ped data.ped data.map --input-map genetic_map_chr1_b36.txt --output-graph shapeit.graph.bin
```

```
SHAPEIT -convert --input-graph shapeit.graph.bin --output-max phased.haps phased.sample
```

Conversion of the pased data into impute format with a script by Daniel Lawson (http://www.maths.bris.ac.uk/~madjl/finestructure/impute2chromopainter.pl.zip):

```
perl impute2chromopainter.pl -f phased.haps genetic_map_chr1_b36.txt CPinput
```

Section C. Chromosome painting with ChromoPainter

ChromoPainter with 990 references and 1 unknown for 1 x 991 matrix (array)

(http://www.maths.bris.ac.uk/~madjl/finestructure/chromopainter_info.html)

```
ChromoPainter -g CPinput.haps -r CPinput.recomrates -a 991 991
```

ChromoPainter with 990 references for 990 x 990 matrix

```
ChromoPainter -g REFinput.haps -r REFinput.recomrates -a 0 0
```

Note1: In order to obtain the REFinput file one needs to repeat all the steps of this section but omitting the data

for the unknown individual.
*Note2: The similarity matrices are in the *chunkcounts* files.*

Section D. Chunkcounts matix manipulations

The 1 x 991 array has only zeros in the last column. This column needs to be removed (remaining columns = 990). The 990 x 990 matrix has a diagonal of zeros. For ease of following manipulation these diagonal values should be replaced with symbols denoting non-applicable such as “NA”.

Both the array and the matrix need to be averaged by each reference group by the column (not taking into account by the NA fields of the matrix). As a result the number of columns drops from 990 to 22 (990/45=22) and each entry in the row is now expressed as its similarity to the group average as opposed to the similarity to each individual in the group.

Because the 990 x 990 matrix is symmetrical with respect to individuals in the columns and rows it also needs to be averaged by each reference group by the row. This reduces its number of rows to 22 as well. The new matrix shows each reference group's similarity to all other reference groups.

The results of these steps are an array of 22 (1 x 22) and a matrix of 22 x 22. The array contains data for the unknown and the matrix contains data for the references.

Both the array and matrix are next scaled so that the average of each row becomes equal to 1.

Note: If you would like additional instructions for file manipulation and handling please consider a) unix commands, b) MS Excel or equivalent, c) www.toomashaller.com/LFE.html, or contact the autor for help.

Section E. MixFit analysis

Download:

MixFit can be downloaded here: www.geenivaramu.ee/en/tools/mixfit
Please note that you can use pre-compiled version of the script, request a specific compilation type for your needs from the author or compile yourself from the source code. If you use the last option, please ensure that you have Qt 4.3 or later (https://www.qt.io/download/) installed on your system.

Usage:

MixFit compares the unknown array of 22 with the reference matrix of 22 x 22. It finds the mix (amalgamate) of three best-fitting reference groups (rows from the 22 x 22 matrix) to report what fraction of each reference group is used to make up the genetic identity of the unknown.

MixFit uses the following tags:

-file: name of the input file (here the array of 22)
-ref: name of the reference file (here the matrix of 22 x 22)
-out: output file name
-delim: matrix/array delimiter; options: “tab” (default), “space”, “colon”, “semicolon”, “comma”, or any freely selected text

-header: whether the input array has a vertical header; options: “no” (default), “yes”
-refheader: whether the input matrix has a vertical header; options: “no” (default), “yes”
-refpops: the number of reference populations (here 22)
-plimit: ancestry fraction (value) under which the component is considered irrelevant and is removed from consideration (in which case less than 3 ancestry components are reported); this can be any number between 0 and 1 (deafault is 0.1)
-step: fraction by which each reference population weight is incremented during the process of best fitting; default = 0.05
-choosebest: allows to fix the identities of some ancestry components before best mix according to the overall similarity between the references and the unknown. For example “-choosebest 1” immediately selects the overall most similar reference population and starts to use this as one of the components by including it in every best fit simulation. Default value is 0 and this generally makes most sense.
-missing: how is missing value denoted, default = “NA”
-a1: when best fit is carried out by systematically varying the ancestry components fluctuations occur between the best and worst fits. The best fits are expressed as minima in the fluctuations. These minima are recorded for candidate selection later in the algorithm. This flag allows one to change the fraction of best fits stored for later candidate selection. Default = 0.1 (meaning that 10% of the minima are considered for compiling the ancestry candidate list).
-a2: each ancestry assignment as it comes out of the -a1 filter is associated with a GOF (goodness of fit) score. These potential assignments are sorted according to the GOF score and only the lowest scores are let pass. This flag determines how many (what fraction) of best assignments pass to the next round where they are averaged to find the 3 top-scoring ancestry components. Default = 0.1 (meaning that 10% of the assignments with the best GOF scores pass).
-a3: in the final simulation the ancestry components are selected but their relative ratios are unknown, so there is one more simulation where the component amounts are systematically varied. The best answer that this step gives is a function of input uncertainty. Therefore MixFit allows the user to average certain number of best solutions. Default = 0.1 (meaning that 10% of the best solutions will be averaged for the very final ancestry component ratios). Note that this number should generally be small.

Minimal example for running Mixfit

MIXFIT -file unknowns.txt -ref references.txt -refpops 22 -out results.txt

Full example for running MixFit

MIXFIT -file unknowns.txt -ref references.txt -refpops 22 -out results.txt -delim space -header yes -refheder yes -plimit 0.1 -step 0.05 -a1 0.3 -a2 0.2 -a3 0.01

Note: The array of 22 values should be arranged in one row (not vertically) in the input file. The orientation is therefore the same as in the 22 x 22 matrix.

Note: The input file can contain more than one array. If more than one individual is analyzed, all the arrays need to be placed under each other row-wise. The result is a non-symmetrical matrix. In this situation MixFit analyzes all individuals automatically.

IMPORTANT: Both the input array and the input matrix have to have ID's in the first column.

Therefore the 22 x 22 matrix and the array of 22 actually have 23 columns.

Section F. Testing

The example files (test_unknowns.txt and test_references.txt) to test MixFit are included in the MixFit download package. Please run this minimal example test to see how MixFit works:

MIXFIT -file test_unknowns.txt -ref test_references.txt -out results.txt -refpops 22

Section G. MixFit output file

MixFit produces output where ancestry components and their values are listed behind each individual ID. Additionally several scores are reported that indicate the quality of the results. Here's an example:

individual1 fractP1: 30.35 fractPcount1: 34 EST 0.436 FIN-S 0.318 LAT 0.244 score: 1.27fractP2: 41.03 fractPcount2: 5194

Below is the meaning of these parameters:

individual1 - the ID of the individual
fractP1: 30.35 – the percent that passed the '-a1' filter
fractPcount1: 34 – the number of values that passed the '-a1' filter
EST 0.436 – an ancestry component assignment and the component value
FIN-S 0.318 – an ancestry component assignment and the component value
LAT 0.244 - an ancestry component assignment and the component value
score: 1.27 – the GOF (goodness of fit score, smaller is better)
fractP2: 41.03 – the percent values that passed the '-a2' filter
fractPcount2: 5194 – the number of values that passed the '-a2' filter

Section H. Computational speed

The computational times of the pipeline vary significantly between different experimental setups such as the size and number of reference groups, the amount of genotype info included etc. We took a very conservative approach at the expense of computational speed in order to achieve maximal assignment quality. We phased each unknown individual's genome data separately against those of the reference individuals. The results would have been quite similar if we had done all phasing in one step. This, however, would have translated into a phasing speed gain of N times where N is the number of unknown individuals. We did not rigorously study the computational time of the entire pipeline as a function of various factors and settings. However, our conservative settings yielded pipeline speeds of several hours per individual. We used a highly parallelizable computational platform.
